# Unresected screen-detected ductal carcinoma in situ: Outcomes of 311 women in the Forget-Me-Not 2 study

**DOI:** 10.1016/j.breast.2022.01.001

**Published:** 2022-01-04

**Authors:** Anthony J. Maxwell, Bridget Hilton, Karen Clements, David Dodwell, Joanne Dulson-Cox, Olive Kearins, Cliona Kirwan, Janet Litherland, Senthurun Mylvaganam, Elena Provenzano, Sarah E. Pinder, Elinor Sawyer, Abeer M. Shaaban, Nisha Sharma, Hilary Stobart, Matthew G. Wallis, Alastair M. Thompson

**Affiliations:** a Nightingale Centre, Wythenshawe Hospital, Manchester University NHS Foundation Trust, Southmoor Road, Manchester, M23 9LT, UK; b Division of Informatics, Imaging & Data Sciences, School of Health Sciences, Faculty of Biology, Medicine and Health, University of Manchester, Manchester, M13 9PT, UK; c Public Health England, 5 St Philip's Place, Birmingham, B3 2PW, UK; d Nuffield Department of Population Health, University of Oxford, Richard Doll Building, Old Road Campus, Oxford, OX3 7LF, UK; e Division of Cancer Sciences, Faculty of Biology, Medicine and Health, University of Manchester, Manchester, M13 9PT, UK; f West of Scotland Breast Screening Centre, Nelson Mandela Place, Glasgow, G2 1QY, UK; g New Cross Hospital, Royal Wolverhampton NHS Trust, Wolverhampton Road, Wolverhampton, WV10 0QP, UK; h Department of Histopathology (Box 235), Addenbrookes Hospital, Hills Road, Cambridge, CB2 0QQ, UK; i Division of Cancer Studies, King's College London, Guy's Hospital, St Thomas Street, London, SE1 9RT, UK; j School of Cancer & Pharmaceutical Sciences, Kings College London, Guy's Cancer Centre, Great Maze Pond, London, SE1 9RT, UK; k Queen Elizabeth Hospital Birmingham and University of Birmingham, Birmingham, B15 2GW, UK; l Leeds Wakefield Breast Screening Service, Seacroft Hospital, York Road, Leeds, LS14 6UH, UK; m Independent Cancer Patients' Voice, 17 Woodbridge Street, London, EC1R 0LL, UK; n Cambridge Breast Unit, Cambridge University Hospitals NHS Foundation Trust, Cambridge & NIHR Cambridge Biomedical Research Centre, Cambridge, CB2 0QQ, UK; o Dan L Duncan Comprehensive Cancer Center, Baylor College of Medicine, Houston, TX, 77030, USA

**Keywords:** Female, Breast, Breast neoplasms, Carcinoma, Intraductal, Noninfiltrating, Mass screening, Retrospective studies, Cohort studies, Registries

## Abstract

**Background and aim:**

The natural history of ductal carcinoma in situ (DCIS) is poorly understood. The aim of this cohort study was to determine the outcomes of women who had no surgery for screen-detected DCIS in the 6 months following diagnosis.

**Methods:**

English breast screening databases were retrospectively searched for women diagnosed with DCIS without invasive cancer at screening and who had no record of surgery within 6 months of diagnosis. These were cross-referenced with cancer registry data. Details of the potentially eligible women were sent to the relevant breast screening units for verification and for completion of data forms detailing clinical, radiological and pathological findings, non-surgical treatment and subsequent clinical course.

**Results:**

Data for 311 eligible women (median age 62 years) were available. 60 women developed invasive cancer, 56 ipsilateral and 4 contralateral. Ipsilateral invasion risk increased approximately linearly with time for at least 10 years. The 10-year cumulative risk of ipsilateral invasion was 9% (95% CI 4–21%), 39% (24–58%) and 36% (24–50%) for low, intermediate and high grade DCIS respectively and was higher in younger women, in those with larger DCIS lesions and in those with microinvasion. Most invasive cancers that developed were grade 2 or 3.

**Conclusion:**

The findings suggest that active surveillance may be a reasonable alternative to surgery in patients with low grade DCIS but that women with intermediate or high grade disease should continue to be offered surgery. This highlights the importance of reproducible grading of DCIS to ensure patients receive appropriate treatment.

## Highlights

• >1/3 women with intermediate/high grade DCIS developed invasive cancer within 10 years.
• Approximately 10% of women with low grade DCIS developed invasion within 10 years.
• Most invasive cancers that developed were grade 2 or 3.
• The risk of invasion was age-dependent and higher in younger women.
• Invasion risk was higher in larger DCIS lesions.

## 1 Introduction

The natural history of ductal carcinoma in situ (DCIS) remains poorly understood and the clinical impact of a diagnosis of DCIS is the subject of considerable debate. Concerns have been expressed about possible overdiagnosis and overtreatment, particularly of what has been termed ‘low risk’ DCIS [1,2]. Randomized trials of active surveillance versus guideline-concordant surgery are currently underway: the LOw Risk dcIS (LORIS) trial in the UK [3], Comparison of Operative to Monitoring and Endocrine Therapy (COMET) in the US [4], LOw Risk Dcis (LORD) in Europe [5] and the LOw Risk Tamoxifen Treatment And surveillance (LORETTA) trial in Japan [6]. In addition, in response to the Cancer Research UK Grand Challenge ‘When is cancer not really cancer’, an international initiative known as PRECISION (PREvent ductal Carcinoma In Situ Invasive Overtreatment Now) is addressing the issues of overdiagnosis and overtreatment via a wide range of approaches [7].

Data from the UK National Health Service Breast Screening Programme (NHSBSP) [8] has demonstrated a significant inverse relationship between DCIS detection rates at screening and subsequent interval cancer rates. In the US National Cancer Database, delay in the surgical treatment of DCIS up to one year after diagnosis was an independent predictor of finding invasive cancer at surgical excision [9]. These effects are probably driven predominantly by the behaviour of high grade DCIS which has a higher rate of proliferation than lower grade disease [10,11].

We have previously published the findings of a Sloane Project sub-study of 89 women in the UK who were diagnosed with DCIS but did not undergo surgical resection or in whom surgery was delayed for at least 12 months (the ‘Forget-Me-Not 1 study’ - FMN1) [12]. This showed a significantly higher rate of progression to invasive cancer in women with high grade DCIS compared to those with lower grades: 14 of 29 women with high grade DCIS developed invasive cancer after a median interval of 38 months but only 3 of 17 women with low grade DCIS developed invasion at 51 months. Progression appeared to be more likely in younger women, in those who had not received endocrine therapy and in those with mammographic microcalcification. While of interest, this was a relatively small and heterogeneous group of mostly older women (median age 75 years), many of whom were diagnosed symptomatically. As the majority of DCIS is diagnosed at screening, the present study focuses exclusively on women with DCIS detected in the English NHSBSP who did not undergo surgery, to determine their long-term outcomes and the risk factors that determine progression to invasive cancer. The English NHSBSP (the other three UK devolved nations have their own screening programmes) currently invites women aged 50–70 for screening every 3 years, although until recently some women aged 47–49 and 71–73 were invited as part of the Age Extension Trial (AGEX) [13] and some women older than 70 have self-referred. Approximately two million women are screened every year [14].

## 2 Materials and methods

This was a retrospective cohort study of women diagnosed with DCIS in the English NHSBSP who had no surgery within 6 months of diagnosis, to determine long term implications of omission of, or delay to, surgery.

### 2.1 Patient identification

The English NHSBSP/Association of Breast Surgery (ABS) National Breast Screening Audit dataset was searched for potentially eligible women who fulfilled all three of the following criteria:

• Ductal carcinoma in situ (DCIS) without concurrent invasive breast cancer (but including DCIS with microinvasion) detected at NHSBSP screening in an English screening unit
• No record of ipsilateral breast surgery within the 183 days (6 months) following the date of the index screening mammogram
• Date of first offered screening appointment (for the episode at which the DCIS diagnosis was made) was between April 1, 2001 and March 31, 2018 inclusive.

Women who had previously had breast cancer (invasive or non-invasive) or previous biopsy-proven atypia were excluded, as were participants in the LORIS study [3].

The resulting list of women was cross-referenced with English National Cancer Registration and Analysis Service (NCRAS) records to exclude women who had a record of surgery within 6 months of the DCIS diagnosis. These were typically women whose surgery was not recorded on the National Breast Screening System (NBSS) because it took place at a site other than the screening centre, usually either another NHS hospital or a private hospital.

### 2.2 Data collection

Data forms for recording radiology, histopathology, treatment and outcome data were developed (Appendix A, Appendix A). Confidential lists of the identified potentially eligible patients were sent to each screening unit with requests to verify the eligibility of individual patients and to complete the data forms. The majority of women only required a single two-page form to be completed. Those who had undergone further biopsy and/or surgery after the initial diagnostic assessment (and six or more months after the reference screening mammogram) required completion of an additional two-page form. Copies of relevant histopathology reports were requested. Radiology and histopathology data were as provided by the individual screening centres (there was no centralised reporting). These were returned to the Sloane Project office at Public Health England (PHE) for database entry and analysis. Cross-checks were made with the NCRAS database to identify any invasive cancers and deaths that had not been identified by the screening units.

Where units did not respond to the data request but women had already been included in our previous study of unresected DCIS [12] or in the main Sloane Project database, we used information from these datasets along with follow-up data from the NCRAS database to include these women in the study.

### 2.3 Data end-points

The duration of follow-up for individual patients was calculated as the time from the positive screening mammogram to the diagnosis of invasive breast cancer. Patients were right censored at the following points:

- surgery for DCIS
- death

If none of these events occurred, the patients were right censored using the following end dates:

- if the unit stated that patient was still alive, the date on which the form was completed
- if the unit did not know the status of the patient, the date the patient was last known to be alive by NCRAS

### 2.4 Statistical analysis

Only univariable analysis was performed due to the relatively small size of the dataset. Comparisons of categorical data were made using Fisher's Exact test where numbers were small, otherwise the Chi-squared test. Continuous variables were assessed by the Mann-Whitney *U* test. Cumulative invasive cancer incidence curves were compared using Kaplan-Meier analysis and the Mantel-Haenszel Log Rank test. The cumulative risks of ipsilateral invasive breast cancer at three time points after the diagnosis of DCIS was calculated using the life table analysis method. Missing data were excluded from the analysis. Analysis was conducted using Stata version 15 (StataCorp LLC, College Station, Texas, USA).

### 2.5 Permissions

This is a retrospective cohort study based on existing data collected under Section 251 of the UK National Health Service Act 2006 and therefore no individual patient consent or ethics committee approval was required. The study was approved by Public Health England's Breast Screening Programme Research Advisory Committee.

## 3 Results

**Fig. 1:**
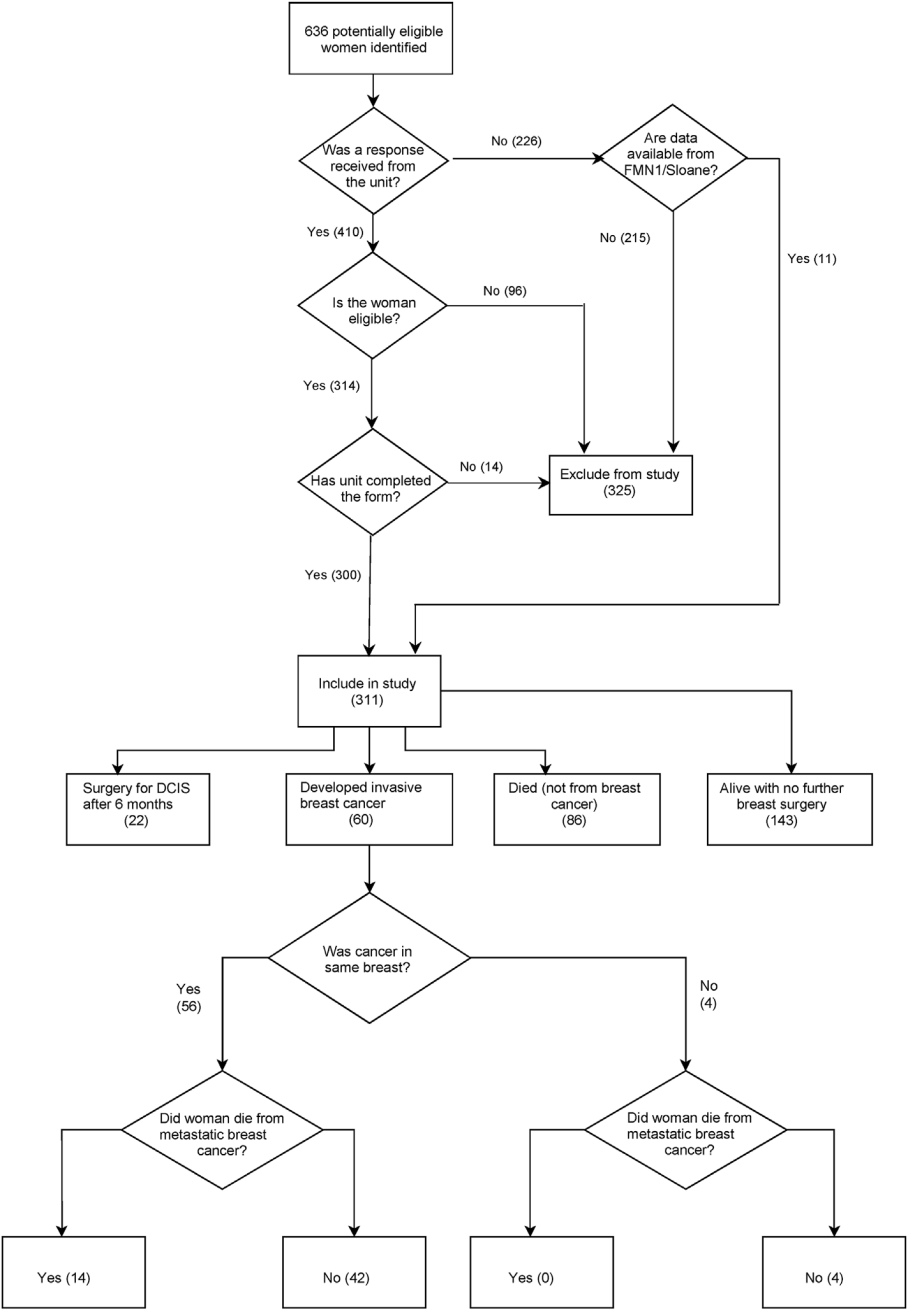
Study flowchart.

There were 636 women who were identified as potentially eligible. Completed forms were received for 410 of these, of whom 110 were excluded (Fig. 1). The remaining 300 women included 32 who had been previously included in the Sloane Project [15] and/or the Forget-Me-Not 1 datasets [12]. A further 11 eligible women, for whom a response was not received, were added from these datasets, giving a total of 311 women. In all, 30 women from the previously published Forget-Me-Not 1 dataset [12] were included, but now with additional follow-up. Data on the eligible women were contributed by 56 screening units (see Acknowledgements).

In order to test for selection bias (possible selective inclusion of women who developed invasive cancer), the proportion of women who developed invasive cancer among the 410 women for whom completed forms were received was compared with that for the 226 women for whom no data were received. These were almost identical at 17% and 16% respectively (71/410 v. 36/226; Chi-square test p = 0.78).

The age range was 47–90 years (interquartile range 54–69; median 62). The primary reasons given for no initial surgery were as follows: 155 women declined (these include 6 who were also classed as unfit for surgery), 86 were unfit for surgery, 20 had other concurrent cancers, 22 had no radiologically visible residual DCIS after biopsy and 28 had other or unknown reasons.

### 3.1 Baseline characteristics

Baseline demographic, imaging and clinical characteristics are shown in Table 1. In summary, there was a slight right-sided DCIS preponderance (52%), with the majority of lesions (56%) involving the upper outer quadrant. Mammographic microcalcification was present in 91% of lesions and was the predominant radiological feature in 83% of women. The most common microcalcification pattern was granular (49%), followed by casting (30%). The median lesion size was 22 mm (range 3–200 mm).

**Table 1:** Baseline radiological and clinical findings.

Feature		Number	% of total
Type of mammogram	Digital	206	66
	Film-screen	79	25
	Not known	26	8
Age at mammogram	<60	130	42
	≥60	181	58
Side	Left	148	48
	Right	163	52
Site^a^	Upper outer quadrant	173	56
	Upper inner quadrant	53	17
	Lower outer quadrant	42	14
	Lower inner quadrant	51	16
	Retroareolar	24	8
	Not known	9	3
Microcalcifications	Present	282	91
	Absent	23	7
	Not known	6	2
Microcalcification pattern	Casting	86	30
	Granular	139	49
	Punctate	42	15
	Other	1	0
	Not known	14	5
Microcalcification distribution	Diffuse	22	8
	Grouped	110	39
	Linear	21	7
	Regional	30	11
	Segmental	42	15
	Not known	57	20
Predominant mammographic feature	Calcification	258	83
	Mass - well defined	10	3
	Mass - ill defined	22	7
	Distortion	5	2
	Spiculate Mass	1	0
	Other	8	3
	Not known	7	2
Maximum lesion size (mm)	1–10	82	26
	11–20	57	18
	21–40	65	21
	41–60	39	13
	61–80	25	8
	>80	20	6
	Not known	23	7
BIRADs breast density	a	21	7
	b	123	40
	c	83	27
	d	16	5
	Not known	68	22

a More than one site can be specified per case.

Breast density was most commonly BI-RADS b (scattered areas of fibroglandular density).

In those in whom clinical examination and ultrasound were performed and the findings known, these were normal or benign in 250/271 women (92%) and 183/253 women (72%) respectively.

Histological diagnosis of DCIS (Table 2) was made on the basis of a 14-gauge core needle biopsy (CNB) in approximately two-thirds of patients, with the majority of the remainder diagnosed on vacuum-assisted biopsy (VAB). Of the 296 for whom the biopsy guidance method was recorded, 241 (81%) were stereotactic, 53 (18%) ultrasound and 2 (1%) freehand. The most common DCIS grade was high grade (123 of the 304 with known grade; 40%). Possible or definite microinvasion was identified in 25 of the 262 (10%) with a known status. Oestrogen receptor (ER) was positive in 115 of the 132 (87%) with a known ER status.

**Table 2:** Baseline DCIS biopsy and histopathological features.

Feature	Number	% of total	% of stated/known
**Biopsy type**			
14 gauge core	204	66	67
Vacuum-assisted	100	32	33
Other	2	1	1
Not stated	5	2	
**DCIS grade**			
High	123	40	40
Intermediate	105	34	35
Low	76	24	25
Not stated	7	2	
**Necrosis**			
Present	89	29	36
Not present	157	50	64
Not stated	65	21	
**Microinvasion**			
Present	10	3	4
Possible	15	5	6
Absent	237	76	90
Not stated	49	16	
**ER status**			
Positive	115	37	87
Negative	17	5	13
Not known	109	35	
Not stated	70	23	
**PR status**			
Positive	42	14	72
Negative	16	5	28
Not known	169	54	
Not stated	84	27	
**HER2 status**			
Positive	4	1	31
Negative	9	3	69
Not known	199	64	
Not stated	99	32	

### 3.2 Non-surgical treatment

Sixty-seven women (22%) were recorded as having been prescribed endocrine therapy (ET), three of whom also had radiotherapy. Two women had vacuum-assisted excision (VAE), one in combination with radiotherapy, and one had radio-frequency ablation. Two women had radiotherapy alone. Overall 2% of women received radiotherapy.

### 3.3 Outcomes

Follow-up from the index screening mammogram was 6–209 months (0.5–17.4 years), with a median of 49 months (4.1 years). Twenty-two women (7%) underwent surgery for DCIS, none of whom had invasive cancer at surgery (median time from diagnosis 24 months; range 9–155 months), 60 (19%) developed invasive breast cancer and 86 (28%) died of other causes.

Of the 60 women with invasive breast cancer, 10 were diagnosed at screening, 24 following GP or outpatient referral, 1 at emergency presentation and 25 were unknown. The invasive cancer was in the same breast as the primary DCIS in 56 and in the contralateral breast in 4. The women who developed ipsilateral invasive breast cancer (iIBC) comprised 28/123 (23%) with high grade DCIS, 21/105 (20%) with intermediate grade DCIS, 5/76 (7%) with low grade DCIS and 2/7 (29%) with an unknown grade of DCIS.

**Fig. 2:**
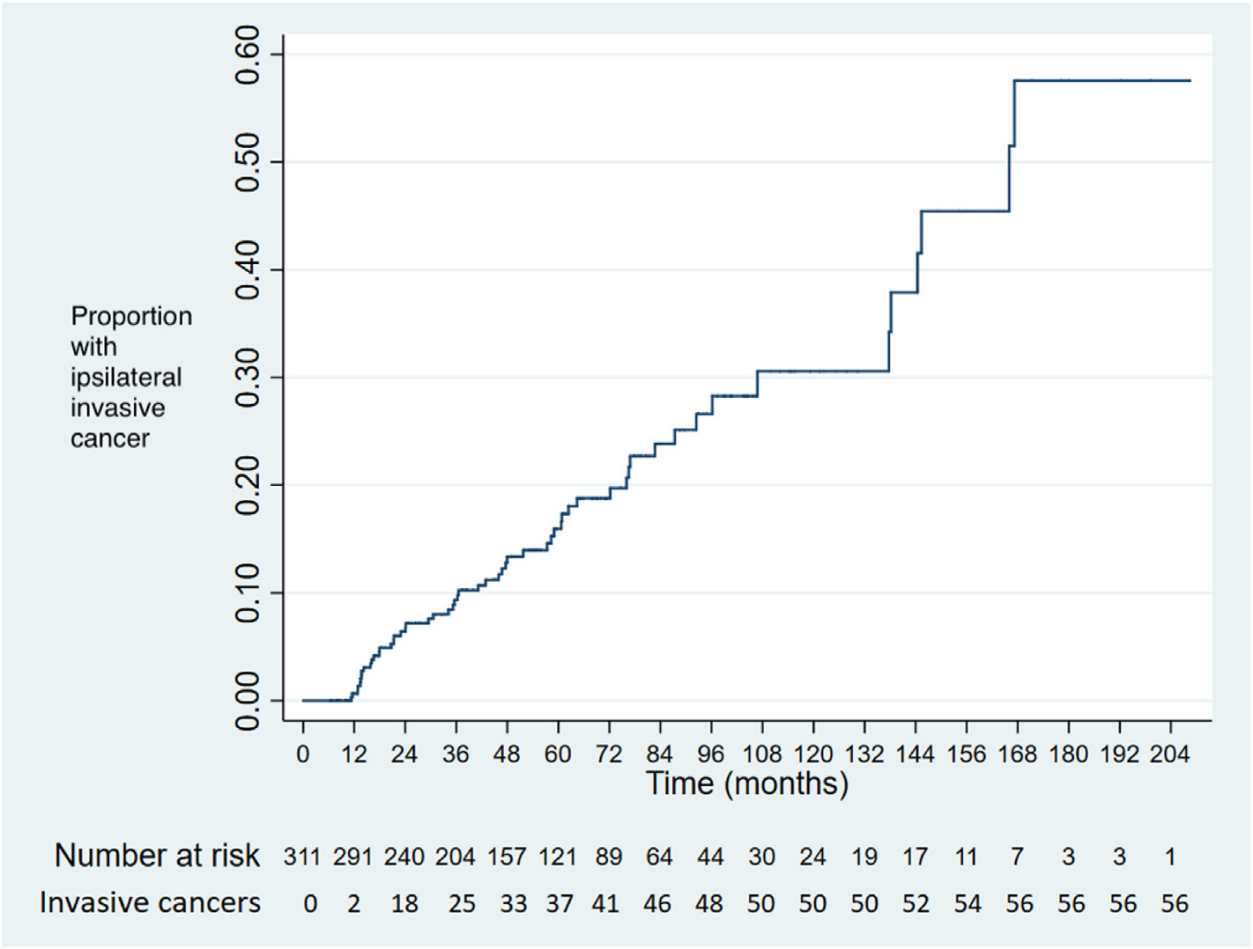
Kaplan-Meier chart showing time to diagnosis of ipsilateral invasive cancer (all cases).

The time to diagnosis of iIBC for all cases is shown in Fig. 2.

**Fig. 3:**
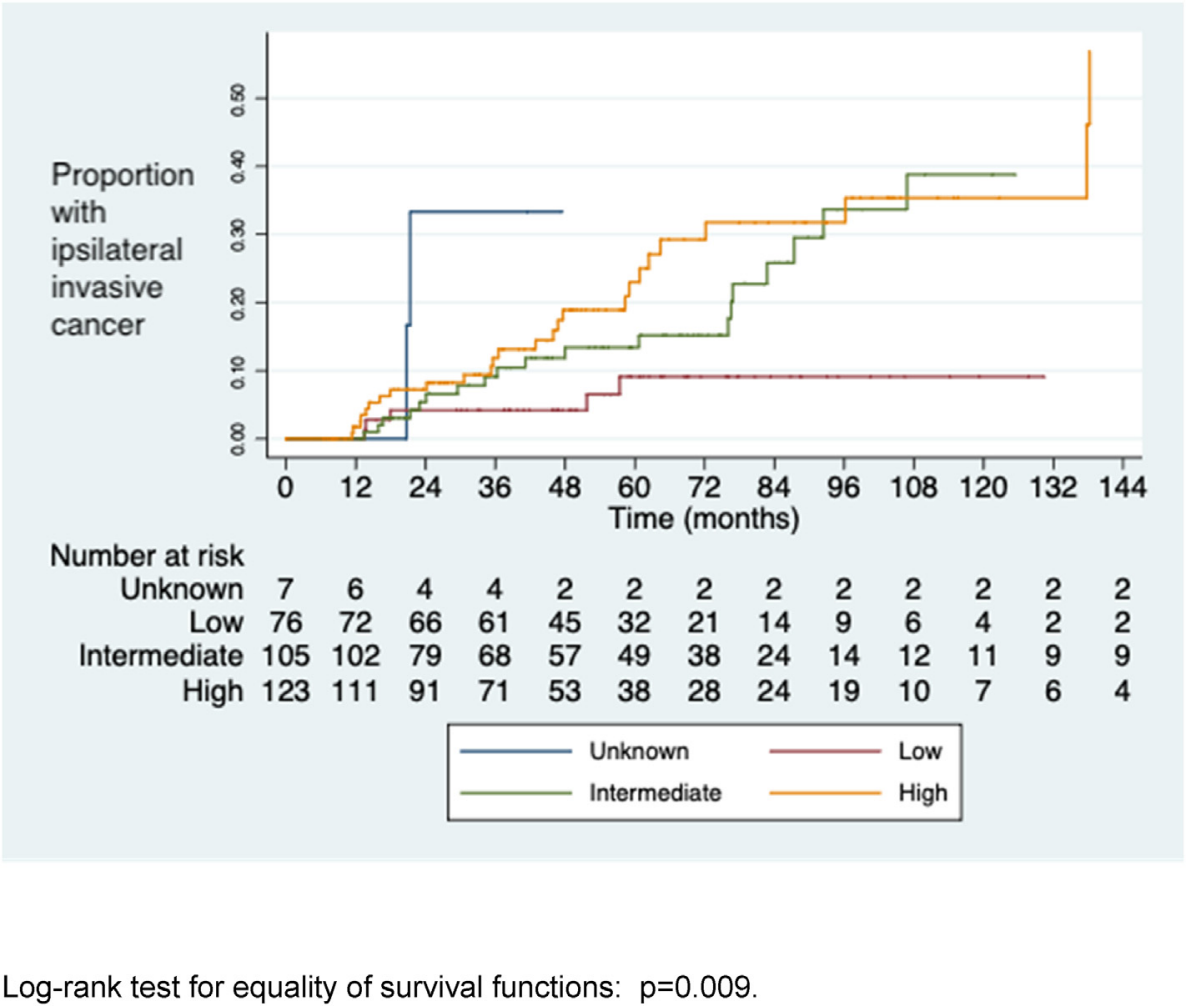
Time to diagnosis of ipsilateral invasive cancer by DCIS grade.

**Fig. 4:**
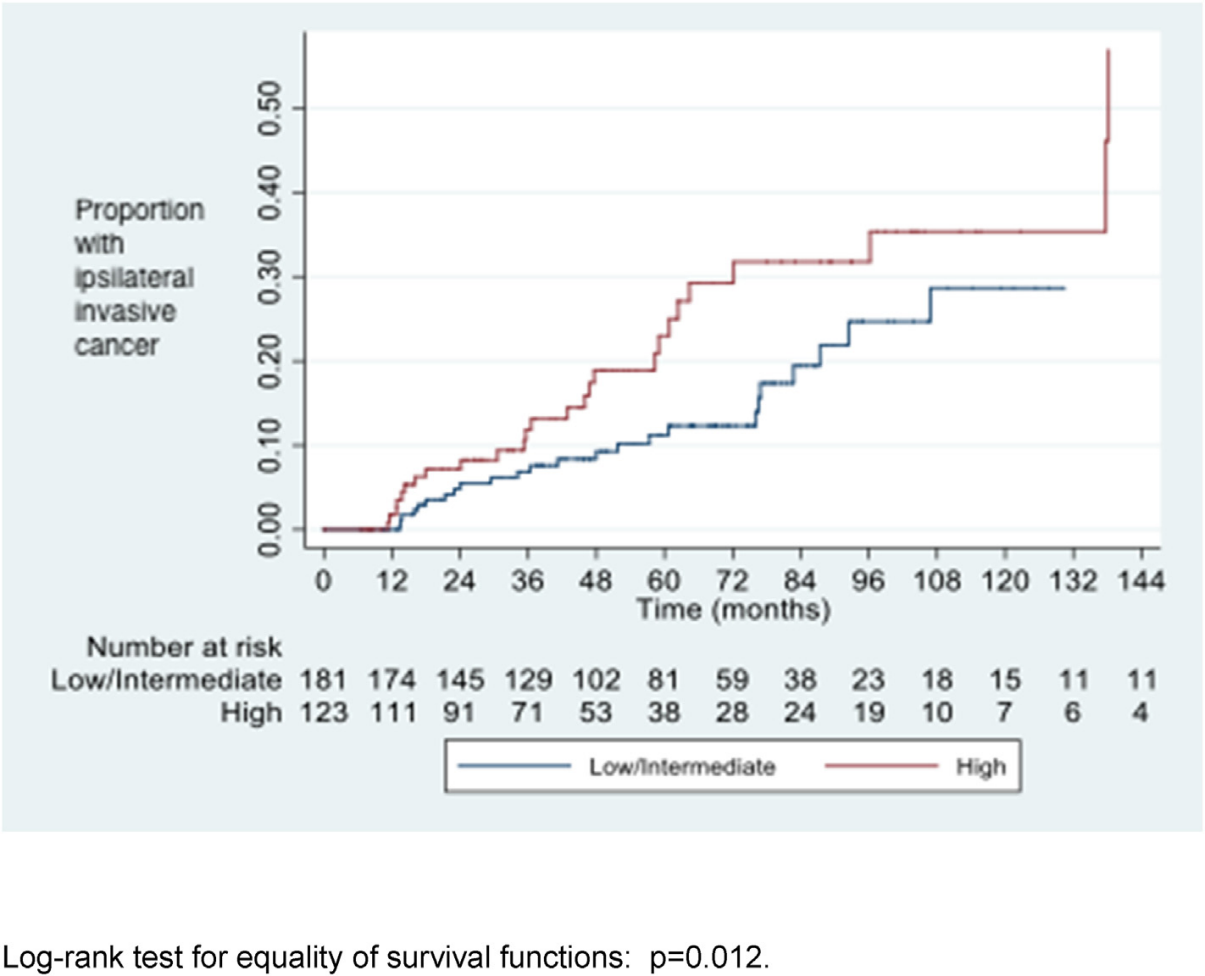
Time to diagnosis of ipsilateral invasive cancer by DCIS grade (high grade v. Intermediate and low).

**Fig. 5:**
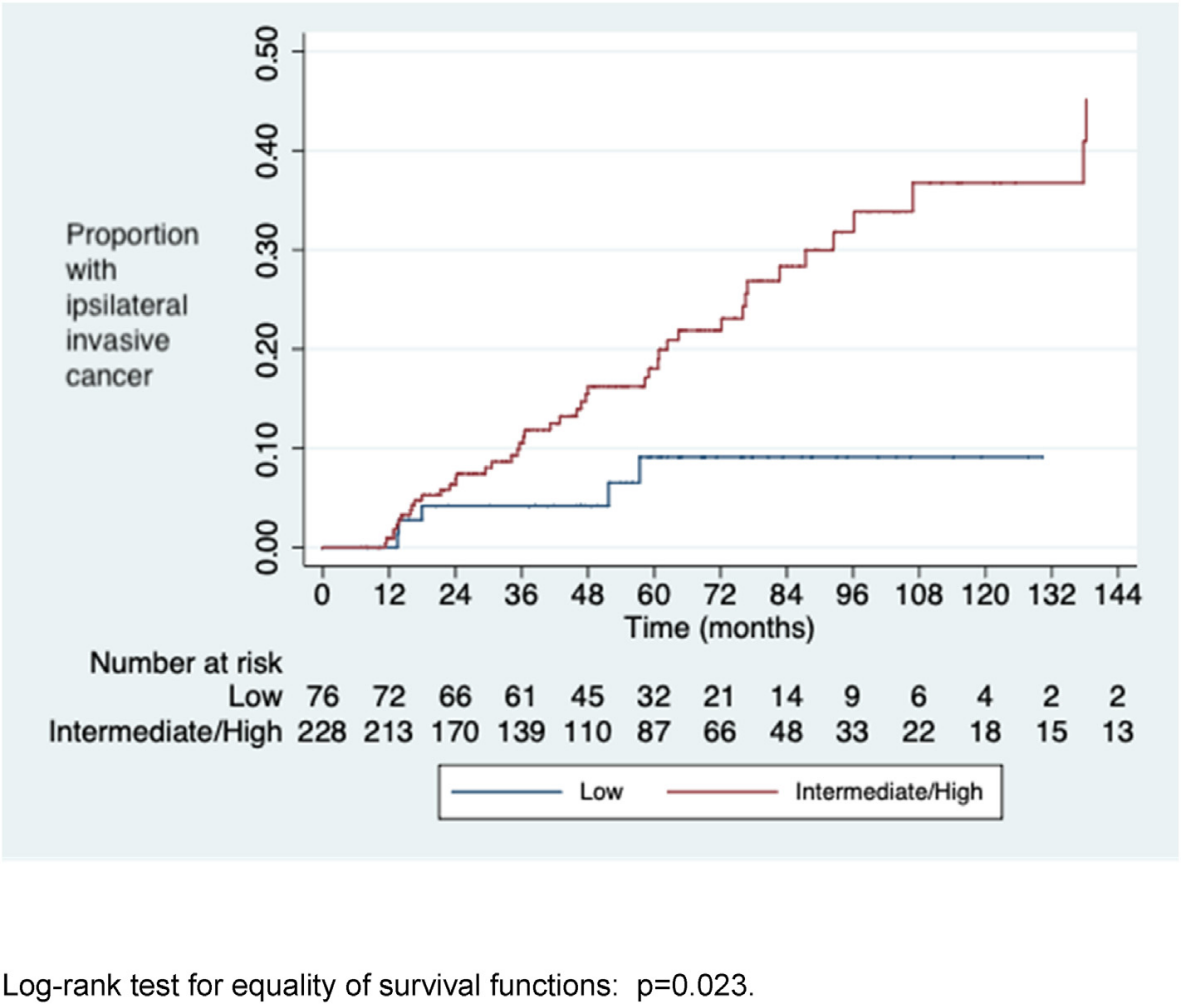
Time to diagnosis of ipsilateral invasive cancer by DCIS grade (high and intermediate grade v. low).

The cumulative risks of iIBC at 5, 8 and 10 years after diagnosis of DCIS are shown in Table 3. Women with high and intermediate grade DCIS were significantly more likely to develop iIBC than those with low grade DCIS (Fig. 3, Fig. 4, Fig. 5). Women who developed iIBC were significantly younger than those who did not, driven primarily by a strong association among those with high grade DCIS. There was no significant difference in age among those with intermediate and low grade DCIS respectively between those who did and did not develop iIBC.

**Table 3:** Development of ipsilateral invasive cancer, DCIS grade, patient age and cumulative risk at 5, 8 and 10 years (life table analysis method).

Grade of DCIS	All cases	Ipsilateral invasion Median age (range) (years)	No ipsilateral invasion	p-value for comparison of ages	5-year risk (95% CI)	8-year risk	10-year risk (95% CI)
	Median age (range) (years)		Median age (range) (years)	(ipsilateral invasion v no ipsilateral invasion)^a^		(95% CI)	
High	63	56	64	0.005	0.23 (0.15–0.34	0.31 (0.22–0.45)	0.36 (0.24–0.50)
(n=123)	(49–86)	(49–71)	(49–86)				
Intermediate	62	59	63	0.29	0.13 (0.07–0.23	0.34 (0.21–0.51)	0.39 (0.24–0.58)
(n=105)	(48–90)	(48–84)	(50–90)				
Low	61	65	60	0.61	0.09 (0.04–0.21	0.09 (0.04–0.21)	0.09 (0.04–0.21)
(n=76)	(47–84)	(50–77)	(47–84)				
Intermediate & low	62	60	62	0.63	0.11 (0.07–0.18)	0.25 (0.16–0.37)	0.28 (0.18–0.43)
(n=181)	(47–90)	(48–84)	(47–90)				
All grades^b^	62	59	63	0.02	0.16 (0.12–0.22)	0.27 (0.20–0.35)	0.31 (0.23–0.40)
(n=311)	(47–90)	(48–84)	(47–90)				

a Mann-Whitney *U* test.

b Includes 7 women with unknown grade.

Of the 262 women with a known microinvasion status, 8/25 (32%) with definite or possible microinvasion were subsequently diagnosed with ipsilateral invasive cancer compared to 38/237 (16%) without microinvasion (p = 0.027).

There was no significant association on univariable analysis between risk of iIBC and microcalcification as the predominant radiological feature (iIBC and microcalcification 47/258 vs iIBC and other radiological feature 7/46; p = 0.83), the presence of histological necrosis (iIBC and necrosis, 14/89 vs iIBC and no necrosis, 31/157; p = 0.49) or the use of ET (iIBC and ET 11/67 vs iIBC and no ET 45/244; p = 0.86).

The median baseline size of DCIS in women who developed iIBC was higher than in women who did not develop iIBC, both overall and in each of the three grade categories (Table 4), reaching statistical significance when all grades were combined.

**Table 4:** Univariable analysis of lesion size (maximum dimension on imaging in mm) and incidence of ipsilateral invasion by DCIS grade (Mann-Whitney *U* test).

DCIS grade	All cases	Invasion		No invasion		p-value
	Median size	Median size	Range	Median size	Range	
High	32	38.5	3–95	29.5	3–162	0.19
Intermediate	20	35	4–80	18	3–121	0.31
Low	16	18	12–50	15	3–200	0.38
All grades	22	37	3–95	20	3–200	0.02

The grade and histological sub-type of the 56 ipsilateral invasive cancers that developed compared to the grade of the original DCIS is shown in Table 5. Forty-eight of these were of ductal/no special type (NST). Of the 51 invasive cancers with a known grade, 46 (90%) were grade 2 or 3.

**Table 5:** Ipsilateral invasive cancer type and grade against original DCIS grade.

Invasive cancer type	Invasive cancer grade	DCIS grade				
		Low	Intermediate	High	Unknown	Total
Ductal/NST	1	1	2	1		4
	2	1	9	10	1	21
	3	3	5	12		20
	not known		1	2		3
Lobular	2		1	2		3
Mucinous	1		1			1
	2		1			1
Papillary	not known			1		1
Unknown	3				1	1
	not known		1			1
**TOTAL**		5	21	28	2	56

Twenty-two women did not undergo initial surgery because the radiologically visible DCIS was removed at needle biopsy (9 with 14-gauge biopsy, 13 at VAB). Of these, one woman later underwent delayed surgery for DCIS and three (14%) developed ipsilateral invasive cancer, of whom two had intermediate grade DCIS and one had high grade DCIS. Median tumour size before biopsy was 7 mm for those who had 14-gauge core biopsy and 5 mm for those who underwent VAB, with median follow-ups of 69 and 48 months respectively.

Table 6 shows the cause of death of the 23 women with invasive breast cancer who died. No significant correlation was found among any of the measured disease or treatment variables between those who developed metastatic breast cancer and those who did not.

**Table 6:** Numbers who died and cause of death among those women who developed invasive breast cancer.

Invasive cancer side	Died	Cause of death		
		Breast cancer	Other cancer	Non-cancer
Ipsilateral (n = 56)	22	14	2	6
Contralateral (n = 4)	1	0	1	0
All (n = 60)	23	14	3	6

## 4 Discussion

This study demonstrates that the risk of developing iIBC in association with unresected DCIS increases with time in an approximately linear fashion, at least for the first 10 years. The risk of iIBC is around 14 times greater than that of contralateral invasive cancer. The risk is higher for those with high and intermediate grade DCIS, who have a slightly greater than 1 in 3 chance of ipsilateral invasion at 10 years, whereas the risk of invasive cancer in those with low grade DCIS is only around 1 in 10 in the same period.

The outcomes for the UK Sloane Project cohort of DCIS patients who had undergone surgery have recently been published [16]. This demonstrates a similar linear increase in ipsilateral invasive recurrences with time in those who had undergone breast conserving surgery (BCS), although the incidence was slightly lower following surgery for high grade DCIS at 4.9% than intermediate/low grade DCIS at 6.7%. This appeared to be due to less frequent use of radiotherapy following BCS for non-high grade DCIS. The incidence of contralateral invasive cancer in that series was 3.5% (325/9191) after a median follow-up of 9.4 years which is consistent with the 1.3% (4/311) after a median follow-up of 4.1 years in the current study. The study by Mannu et al. [17] of over 35,000 women with screen-detected DCIS, some followed up for over 20 years, also shows a progressive linear increase in the incidence of invasive breast cancer and breast cancer mortality.

The risk of developing invasion appears to be greater for younger women, at least for those with high grade DCIS. Although this is on univariable analysis, the age distribution profiles for high, intermediate and low grade DCIS are similar and this is therefore likely to be a true effect. This age-dependent risk was also demonstrated in our previous study [12] and is consistent with the higher risk of invasive local recurrence in young women following wide local excision of DCIS [18].

The finding of a higher likelihood of the subsequent diagnosis of invasive cancer in those with definite or possible microinvasion (invasive foci measuring ≤1 mm) may in some cases be due to under-diagnosis of larger invasive foci because of the limited amount of tissue sampled in a needle biopsy. It may also be a reflection of biologically more aggressive disease.

Although endocrine therapy (ET) would be expected to reduce the risk of developing invasive cancer, only 64 women in the study were recorded as having been prescribed ET. There is no record of compliance with treatment and no association between the use of ET and the occurrence of invasive cancer was demonstrated. This contrasts to our previous study [12] where ET was associated with a halving of the risk of invasion. The current finding may be due to the low numbers or to lack of compliance in a patient cohort that is already having non-standard treatment.

The iIBCs that developed were predominantly of ductal/NST subtype, although at least six were of special type. The complexities of development of different histological sub-types of invasive breast cancer is poorly understood [19]. DCIS is sometimes the only precursor seen in association with invasive lobular carcinoma in routine histological practice, and conversely lobular carcinoma in situ (LCIS) may be the only in situ lesion that is seen in an area of invasive carcinoma of ductal/no special type. DCIS may also be a potential marker for the development of cancers not directly arising from the in situ lesion. Whilst the risk of contralateral breast cancer is well recognised for patients with LCIS, there is also a higher risk of contralateral disease in patients with DCIS compared to women without a history of breast cancer [20]. We do not have data allowing us to compare the site of the DCIS in the breast with the location of the subsequent ipsilateral invasive cancers and the small number of contralateral cancers (four) is insufficient to compare the risk of contralateral disease in our study population with that of the general population.

At least 40 (82%) of the iIBCs arising in the 49 women with high or intermediate grade DCIS were grade 2 or 3. Only five iIBCs developed in association with low grade DCIS and these were spread across all grades of invasive disease. Overall, only 10% of the invasive cancers with a known grade were grade 1. This is very similar to the grade profile of iIBC recurrences for the English Sloane cohort of 9191 women with screen detected DCIS where 9% of subsequent invasive cancers were grade 1, 46% were grade 2 and 29% were grade 3 (15% were of unknown grade) [16].

The incidence of iIBC in the women who did not undergo initial surgery because of removal of the radiologically visible DCIS at biopsy (14% after a median follow-up of 57 months) is similar to that in the study group overall. Although the numbers are very small (3/22), this does suggest that simple percutaneous removal of radiologically visible disease is unlikely to be an adequate treatment strategy.

Fourteen of the 22 women who developed iIBC died of the disease, underlining the importance of detection and optimum treatment of DCIS.

DCIS is a relatively common condition, affecting approximately 4000 women per year in England and accounting for 20% of screen-detected cancers [21]. However, despite its frequency, the risks of progression to invasive cancer have been the subject of considerable debate. A recent article by Heller and colleagues [22] provides a useful summary of some of the published evidence regarding progression, including discussion of animal models, biopsy specimen review of ‘missed’ DCIS cases, population-based series and modelling studies. They also reviewed the current and proposed trials of active surveillance of DCIS. Not included in their review was a study published in 2019 by Ryser and colleagues [23] who examined the outcomes of 1286 patients in the US National Cancer Institute's Surveillance, Epidemiology, and End Results (SEER) database with DCIS who did not undergo locoregional treatment. This reported an overall net 10-year risk of iIBC of 12% (95% CI 10–15%). The risk in those with high grade disease was 18% (12–25%) and 12% (9–17%) in those with non-high grade disease. These figures contrast markedly with our findings which show a substantially higher 10-year risk of invasion of 31% overall, 36% for high grade disease and 28% for non-high grade disease. These differences are hard to fully explain but may in part be due to incomplete ascertainment of invasive disease in the US study, as women who were diagnosed with invasion outside the registry area where the DCIS was recorded were not included, whereas in our study national cancer registry data were used and ascertainment of invasive disease is more likely to be complete. Also, a greater proportion of women in the SEER study may have received ET, shown to slow progression in our previous study^12^. An additional factor may be differences in pathological grading, which is recognised to be subject to significant inter-observer variation [[24], [25], [26]]. This may also, at least in part, explain why intermediate grade DCIS appears to behave more like high grade disease in this English study whereas its behaviour is more like that of low grade disease in the USA.

The study has a number of limitations. It is a relatively small retrospective study and consequently multivariable analysis was not performed. Data forms were only received for 410 of the 636 (64%) potentially eligible women who were identified from national data but the similar proportion of women who developed invasive cancer in the groups for whom forms were and were not received respectively suggests that there is no reporting bias. It is likely that some women already had invasive cancer at the time the DCIS was diagnosed as both 14-gauge core biopsy and vacuum-assisted biopsy have significant under-estimation rates [27,28], although this does reflect the ‘real world’ situation where management decisions are made on histological analysis of image-guided tissue samples. A few of the 22 women who were reported as having had the radiologically visible DCIS removed at biopsy may have had the disease removed completely, and there may have been under-diagnosis or under-reporting of subsequent invasive cancer among the women who did not undergo initial surgery because of severe comorbidities. Both of these latter factors may have slightly reduced the risk of subsequent invasive cancer in the study group as a whole. The study includes 30 women who were in our previously published series of 89 women [12]. These comprise fewer than 10% of the current study participants but are included as they add value by virtue of their extended follow-up.

## 5 Conclusions

This study is of importance as it includes a unique cohort of patients for whom detailed data are available that provide further insights into the natural history of DCIS and inform management strategy. It indicates that approximately one in three women with untreated high or intermediate grade screen-detected DCIS will develop ipsilateral invasive breast cancer within ten years of diagnosis and that the invasive cancers that develop are likely to be grade 2 or 3. The corresponding risk of developing invasive cancer in those with low grade DCIS, however, is only approximately one in ten. As approximately 90% of women with screen-detected DCIS have intermediate or high grade disease (with the accompanying risk of invasive cancer if untreated), this study emphasises the importance of DCIS detection at screening and its role in reducing breast cancer mortality. The risk of invasion is higher in those who are younger, in those with larger DCIS lesions and in those with definite or possible microinvasion on needle biopsy. The findings suggest that those with high and intermediate grade DCIS and probably those with microinvasion should continue to be offered surgery. For those with low grade DCIS there is a need for shared decision-making in the choice of surgery or active surveillance based on a discussion of the risks and benefits of the options as currently understood and in the light of the low reproducibility of DCIS grading.

## Author contributions

**Anthony J Maxwell:** conceptualisation, funding acquisition, investigation, methodology, vizualisation, writing - original draft. **Bridget Hilton:** data curation, formal analysis, investigation, methodology, project administration, resources, vizualisation, writing - review and editing. **Karen Clements:** data curation, formal analysis, funding acquisition, investigation, methodology, project administration, resources, supervision, writing - review and editing. **David Dodwell:** writing - review and editing. **Joanne Dulson-Cox:** data curation, project administration. **Olive Kearins:** writing - review and editing. **Cliona Kirwan:** methodology, writing - review and editing. **Janet Litherland:** methodology, writing - review and editing. **Senthurun Mylvaganam:** writing - review and editing. **Elena Provenzano:** methodology, writing - review and editing. **Sarah E Pinder:** methodology, writing - review and editing. **Elinor Sawyer:** writing - review and editing. **Abeer M Shaaban:** writing - review and editing. **Nisha Sharma:** methodology, writing - review and editing. **Hilary Stobart:** writing - review and editing. **Matthew G Wallis:** methodology, writing - review and editing. **Alastair M Thompson:** methodology, supervision, writing - review and editing.

## Funding

This study was funded with a grant from Prevent Breast Cancer (ref. GA19-003) - www.preventbreastcancer.org.uk.

The work was supported by the 10.13039/100014653NIHR Manchester Biomedical Research Centre (ref. IS-BRC-1215-20,007) (AJM and CK) and the 10.13039/501100018956NIHR Cambridge Biomedical Research Centre (ref. IS-BRC-1215-20,014) (MW and EP). The views expressed are those of the authors and not necessarily those of the 10.13039/501100000272NIHR or the 10.13039/501100000276Department of Health and Social Care.

KC is part of the Cancer Grand Challenges PRECISION team, funded by 10.13039/501100000289Cancer Research UK and 10.13039/501100004622KWF Kankerbestrijding (ref. C38317/A24043).

DD is funded by 10.13039/501100000289Cancer Research UK (ref. C8225/A21133).

The following English breast screening services contributed to this study:

Avon

Beds & Herts

Bolton

Brighton

Cambridge & Huntingdon

Central and East London

Chelmsford & Colchester

City, Sandwell & Walsall

Cornwall

Derby & Burton

Doncaster & Bassetlaw

Dorset

Dudley & Wolverhampton

East Cheshire & Stockport

East Lancashire

East Suffolk

Gateshead

Guildford

Hereford & Worcester

Humberside

Kent (Canterbury)

Kent (Maidstone)

Kent (Medway)

Leeds Wakefield

Leicestershire & Rutland

Lincolnshire

Liverpool

Manchester

Milton Keynes

Newcastle

Northampton

North Derbyshire & Chesterfield

North Lancashire & South Cumbria

North London

North & Mid Hants

North Midlands

North Yorkshire

Nottingham

Oxford

Peterborough

Portsmouth

Southampton & Salisbury

Sheffield

Shropshire

Somerset

South Birmingham

South Devon

South East London

South Essex

South West London

Warrington, Halton, St Helens & Knowsley

Warwickshire, Solihull & Coventry

West Devon

West Sussex

Wiltshire

Wirral

## Declaration of competing interest

The authors declare that they have no known competing financial interests or personal relationships that could have appeared to influence the work reported in this paper.

## Footnotes

Appendix A Supplementary data to this article can be found online at https://doi.org/mmcdoino.
