# Supplementary material for: Unresected screen-detected ductal carcinoma in situ: Outcomes of 311 women in the Forget-Me-Not 2 study
Source: Breast. 2022 Jan 4;61:145–55. doi: 10.1016/j.breast.2022.01.001 (PMC8753270; doi:10.1016/j.breast.2022.01.001)
Supplement: Multimedia component 1 [file mmc1.pdf]

SLOANE PROJECT QUESTIONNAIRE FOR PATIENTS WITH NO OR DELAYED (>6 MONTHS) SURGICAL TREATMENT OF BIOPSY-PROVEN SCREEN-DETECTED DCIS: FORM 1 (DIAGNOSIS AND FOLLOW-UP)

Please return completed forms to:
Karen Clements, Breast Cancer Research Manager, Public Health England Screening, 5 St Philip's Place, Birmingham, B3 2PW or email to phe.sloaneproject@nhs.net

NHS number: Sx no: DoB:

Treating hospital ☐ Hospital number ☐

Form completed by ☐ Date of completion ☐

Email ☐

ELIGIBILITY

Has the patient had resection of the DCIS within 6 months of the abnormal screening mammogram? No ☐ Yes ☐

Is the patient a randomised participant in the LORIS trial? No ☐ Yes ☐

Does another exclusion criterion apply? No ☐ Yes ☐

If answer is Yes to any question, please give details overleaf & return form. Otherwise complete rest of form.

ASSESSMENT FINDINGS

Mammography

Date of screening mammogram ____________

Digital ☐ Film/screen ☐

Side: Right ☐ Left ☐

Site: *(tick all that apply)*
UOQ ☐
UIQ ☐
LOQ ☐
LIQ ☐
Retroareolar ☐

Does the lesion contain microcalcification?
Yes ☐ No ☐

If yes, most suspicious pattern: *(tick one)*
Casting ☐
Granular ☐
Punctate ☐

Distribution: *(tick one)*
Diffuse ☐
Regional ☐
Grouped ☐
Linear ☐
Segmental ☐

Predominant mammographic feature: *(tick one)*
Calcification ☐
Mass – well-defined ☐
Mass – ill-defined ☐
Distortion ☐
Spiculate mass ☐
None of above – state ____________

Maximum size of lesion ________ mm

BI-RADS breast density: *(tick one)*
a ☐
b ☐
c ☐
d ☐

Clinical examination

P score (1-5) _____
Not performed ☐
Not known ☐

Ultrasound

U score (1-5) _____
Not performed ☐
Not known ☐

Biopsy

Procedure giving definitive DCIS diagnosis:
VAB ☐ Gauge ________
14G core ☐
Other ☐ State ________

Mode:
Stereo/tomo ☐
Ultrasound ☐
Freehand ☐

Number of cores ________

SLOANE PROJECT QUESTIONNAIRE FOR PATIENTS WITH NO OR DELAYED (>6 MONTHS) SURGICAL TREATMENT OF BIOPSY-PROVEN SCREEN-DETECTED DCIS: FORM 1 (DIAGNOSIS AND FOLLOW-UP)

DCIS histology on core biopsy / VAB

Laboratory number ______________________

Nuclear grade (highest present):
- Low ☐
- Intermediate ☐
- High ☐

Necrosis:
- Yes ☐
- No ☐

Microinvasion:
- Yes ☐
- Possible ☐
- No ☐

Receptor status:

**ER**	Positive	☐	Score ________
	Negative	☐	
	Not known	☐	
**PR**	Positive	☐	Score ________
	Negative	☐	
	Not known	☐	
**HER2**	Positive	☐	Score ________
	Negative	☐	
	Not known	☐	

Please send copy of histopathology report with form

Reason for no initial surgery for DCIS

- Patient declined ☐
- Unfit for surgery ☐
- Other ☐

State:

Non-surgical treatment of DCIS

- None ☐
- Tamoxifen ☐ *(tick all that apply)*
- Aromatase inhibitor ☐
- Raloxifene ☐
- Radiotherapy ☐
- Other ☐

State:

Please give details of duration etc. below

FOLLOW-UP

Is the patient still alive?
- Yes ☐
- No ☐
- Not known ☐

If No, please state:

Date of death ______________________

Cause of death ______________________

Has the patient undergone further needle biopsy or surgery on either breast?
- Yes ☐
- No ☐

If yes, please give details on FORM 2

If no further biopsy or surgery:

Date of last mammogram ________________

Max. size of DCIS __________ mm

Any radiological evidence of invasive disease or new radiological abnormality?
- Yes ☐
- No ☐

If yes, please give details under ADDITIONAL INFORMATION

ADDITIONAL INFORMATION
