# Supplementary material for: Unresected screen-detected ductal carcinoma in situ: Outcomes of 311 women in the Forget-Me-Not 2 study
Source: Breast. 2022 Jan 4;61:145–55. doi: 10.1016/j.breast.2022.01.001 (PMC8753270; doi:10.1016/j.breast.2022.01.001)
Supplement: Multimedia component 2 [file mmc2.pdf]

SLOANE PROJECT QUESTIONNAIRE FOR PATIENTS WITH NO OR DELAYED (>6 MONTHS) SURGICAL TREATMENT OF BIOPSY-PROVEN SCREEN-DETECTED DCIS: FORM 2 (FURTHER BIOPSY OR SURGERY)

Please return completed forms to:
Karen Clements, Breast Cancer Research Manager, Public Health England Screening, 5 St Philip's Place, Birmingham, B3 2PW or email to phe.sloaneproject@nhs.net

NHS number: Sx no: DoB:

Treating hospital ☐ Hospital number ☐

Form completed by ☐ Date of completion ☐

Email ☐

REASON FOR FURTHER BIOPSY OR SURGERY

Clinical change ☐
Imaging change ☐
Patient choice ☐
Other ☐ state ______________
On endocrine treatment at time of decision?
Yes ☐ No ☐

Site: At known DCIS ☐
New lesion –
same breast, different site ☐
opposite breast ☐
If >1 changed/new lesion biopsied please complete separate form for each and state number ________

REASSESSMENT AND BIOPSY

Mammography

Date of mammogram _____________

Digital ☐ Film/screen ☐

Side: Right ☐ Left ☐

Site: UOQ ☐ *(tick all that apply)*
UIQ ☐
LOQ ☐
LIQ ☐
Retroareolar ☐

Does the lesion contain microcalcification?
Yes ☐ No ☐

If yes, most suspicious pattern: *(tick one)*
Casting ☐
Granular ☐
Punctate ☐

Distribution: *(tick one)*
Diffuse ☐
Regional ☐
Grouped ☐
Linear ☐
Segmental ☐

Predominant/new mammographic feature: *(tick one)*
Calcification ☐
Mass – well-defined ☐
Mass – ill-defined ☐
Distortion ☐
Spiculate mass ☐
None of above – state ________________

Maximum size of lesion __________ mm

BI-RADS breast density: *(tick one)*
a ☐
b ☐
c ☐
d ☐

Clinical examination

P score (1-5) _____
Not performed ☐
Not known ☐

Ultrasound

U score (1-5) _____
Not performed ☐
Not known ☐

Biopsy

Date of biopsy ______________________

Procedure giving definitive diagnosis:
VAB ☐ Gauge _________
14G core ☐
Other ☐ State _________

Mode:
Stereo/tomo ☐
Ultrasound ☐
Freehand ☐

No. of cores _________

SLOANE PROJECT QUESTIONNAIRE FOR PATIENTS WITH NO OR DELAYED (>6 MONTHS) SURGICAL TREATMENT OF BIOPSY-PROVEN SCREEN-DETECTED DCIS: FORM 2 (FURTHER BIOPSY OR SURGERY)

Histology on core biopsy / VAB

Laboratory number ____________________

DCIS Yes ☐ No ☐

Nuclear grade: Low ☐ Intermediate ☐ High ☐

Necrosis: Yes ☐ No ☐

Microinvasion: Yes ☐ Possible ☐ No ☐

Invasive cancer Yes ☐ Possible ☐ No ☐

Invasive cancer type ____________________

Invasive grade: 1 ☐ 2 ☐ 3 ☐ nk ☐

Receptor status (of invasive cancer if present, otherwise DCIS):

ER Positive ☐ Negative ☐ Not known ☐ | Score ________

PR Positive ☐ Negative ☐ Not known ☐ | Score ________

HER2 Positive ☐ Negative ☐ Not known ☐ | Score ________

Please send copy of histopathology report with form

SURGERY

No surgery ☐
Mastectomy ☐
WLE ☐

Other ☐ state ____________________

Date of surgery ____________________

Surgical pathology

Laboratory number: ____________________

DCIS Yes ☐ No ☐

Nuclear grade (highest present):
Low ☐
Intermediate ☐
High ☐
not known ☐

Invasive cancer Yes ☐ No ☐

Type: Ductal/NST ☐
Lobular ☐
Tubular/cribriform ☐
Mucinous ☐
Medullary-like ☐
Mixed ☐
state types ________________

Other ☐
state ________________

Invasive grade: 1 ☐ 2 ☐ 3 ☐ nk ☐

Nodes: no. examined ________
no. with macromets ________
no. with micromets ________
no. with ITCs ________

Size Total (DCIS +/- invasive) ________ mm
Invasive component ________ mm

Receptor status (of invasive cancer if present, otherwise DCIS)

ER Positive ☐ Negative ☐ Not known ☐ | Score ________

PR Positive ☐ Negative ☐ Not known ☐ | Score ________

HER2 Positive ☐ Negative ☐ Not known ☐ | Score ________

Ki67 Score ________

Please send copy of histopathology report with form

ADDITIONAL INFORMATION
